# Stereoselective Fluorescence Quenching in the Electron Transfer Photooxidation of Nucleobase-Related Azetidines by Cyanoaromatics

**DOI:** 10.3390/molecules21121683

**Published:** 2016-12-07

**Authors:** Ana B. Fraga-Timiraos, Gemma M. Rodríguez-Muñiz, Vicente Peiro-Penalba, Miguel A. Miranda, Virginie Lhiaubet-Vallet

**Affiliations:** Instituto Mixto de Tecnología Química (UPV-CSIC), Universitat Politècnica de València—Consejo Superior de Investigaciones Científicas, Avda de los Naranjos s/n, 46022 Valencia, Spain; anfrati@itq.upv.es (A.B.F.-T.); gemrodmu@itq.upv.es (G.M.R.-M.); vipeipe@etsii.upv.es (V.P.-P.)

**Keywords:** DNA repair, energy and charge transfer, nucleobase analogues, photolyase, redox potential

## Abstract

Electron transfer involving nucleic acids and their derivatives is an important field in bioorganic chemistry, specifically in connection with its role in the photo-driven DNA damage and repair. Four-membered ring heterocyclic oxetanes and azetidines have been claimed to be the intermediates involved in the repair of DNA (6-4) photoproduct by photolyase. In this context, we examine here the redox properties of the two azetidine isomers obtained from photocycloaddition between 6-aza-1,3-dimethyluracil and cyclohexene. Steady-state and time-resolved fluorescence experiments using a series of photoreductants and photooxidants have been run to evaluate the efficiency of the electron transfer process. Analysis of the obtained quenching kinetics shows that the azetidine compounds can act as electron donors. Additionally, it appears that the *cis* isomer is more easily oxidized than its *trans* counterpart. This result is in agreement with electrochemical studies performed on both azetidine derivatives.

## 1. Introduction

Electron transfer involving nucleic acids and their derivatives has been the subject of extensive studies in bioorganic chemistry, primarily because of its role in DNA damage and repair, but also in connection with the possible behavior of DNA as a molecular wire [1,2,3,4,5,6,7,8,9]. Last year, the Nobel Prize in Chemistry awarded for “the mechanistic studies of DNA repair” has emphasized, among others, the importance of photolyase enzymes [10]. They specifically repair bipyrimidine lesions, i.e., cyclobutane pyrimidine dimers (CPD) and (6-4) photoproducts, through a photochemically induced electron transfer from the catalytic flavin-adenosine cofactor to the lesion or to an oxetane/azetidine intermediate [5,11]. Although photorepair in Nature involves a reductive mechanism, the oxidative pathway is also relevant, particularly for long range mediated charge transport [1,12,13]. In this context, the photo-driven cycloreversion of CPD has been addressed through the use of photosensitized reductive or oxidative processes [12,14,15]. Moreover, radical ionic heterocycloreversion of 4-membered rings has emerged as an active field in the last two decades [8,9,16,17,18,19]. Oxetanes have been thoroughly investigated through both oxidative and reductive splitting; by contrast, little is known about thietanes and azetidines [9,16]. The latter are particularly relevant to the understanding of (6-4) photoproduct repair by photolyases at thymine-cytosine sequences. In a recent article, we have designed a model system obtained through photocycloaddition between thymine and 6-azauracil, demonstrating that photosensitizers with potential in the singlet excited state ranging from −3.3 V to −2.1 V (vs. SCE) are appropriate photoreductants [20]. Interestingly, oxidative electron transfer has only been investigated with triphenylazetidines, which are structurally unrelated to pyrimidine bases [21].

**Scheme 1:**
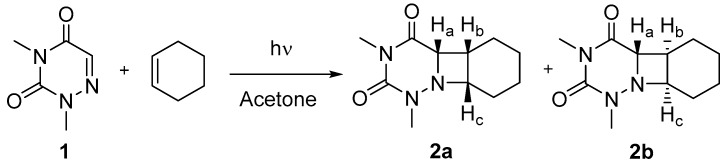
Synthesis of *cis* isomer **2a** and *trans* isomer **2b** from 6-aza-1,3-dimethyluracil (**1**).

**Figure 1:**
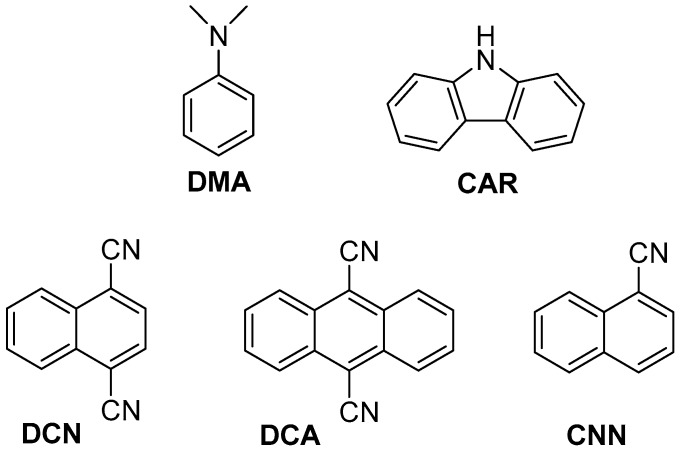
Structure of the used photosensitizers (PS).

With this background, the two azetidine isomers **2a** and **2b** were synthesized and used as substrates for investigating the initial step in reductive and oxidative electron transfer (Scheme 1). This process was studied by steady-state fluorescence using a series of photosensitizers (PS, Figure 1). Both electron donor and acceptor sensitizers were selected covering a large range of singlet excited state redox properties. They are namely *N*,*N*-dimethylaniline (DMA) and carbazole (CAR) for the photoreductants and 1,4-dicyanonaphthalene (DCN), 9,10-dicyanoanthracene (DCA) and 1-cyanonaphthalene (CNN) for the photooxidants (Table 1) [20,22]. Interestingly, and in contrast with our previous results on the thymine/azauracil dimers, the photooxidative process was more efficient. Moreover, a stereoselectivity in the fluorescence quenching was observed between *cis* and *trans* isomers.

## 2. Results

### 2.1. Synthesis

The synthesis of the azetidines **2a** and **2b** (Scheme 1) is described in details in the experimental section. Briefly, 6-aza-1,3-dimethyluracil (see NMR spectra in Figure S1), obtained from 6-azauracil through a known procedure [23], was irradiated (at λ > 290 nm) in the presence of cyclohexene using acetone as photosensitizer. The two isomers formed were separated by liquid chromatography. Their stereochemistry was determined from NMR analysis i.e., ^1^H-, ^13^C- spectra as well as HMQC and NOESY 2D experiments (see Figures S2–S5). A tentative assignment was proposed from the ^1^H-NMR spectra because, in the case of the *cis* isomer, the H_a_, H_b_ and H_c_ protons are located on the same side of the azetidine ring. Thus, H_a_ should be observed as a doublet as it is coupled to H_b_. By contrast, in the *trans* isomer, H_a_ does not interact with the other two protons of the ring, and thus appears as a singlet at ca. 4.0 ppm. However, this signal cannot be observed clearly as it overlaps with H_c_. The NOESY 2D NMR spectrum of **2a** is in agreement with the proposed *cis* configuration as an interaction was observed between the H_a_ and H_b_ protons located at 4.33 and 4.01 ppm, respectively (see Figure S4). Preliminary experiments were performed to evaluate the UVA photoreactivity of **2a** and **2b** under reductive or oxidative conditions. Both isomers were consumed faster in the presence of photooxidant; moreover, it is noteworthy that the *cis* isomer **2a** was photodecomposed more efficiently than its *trans* counterpart **2b**.

### 2.2. Fluorescence Experiments

**Figure 2:**
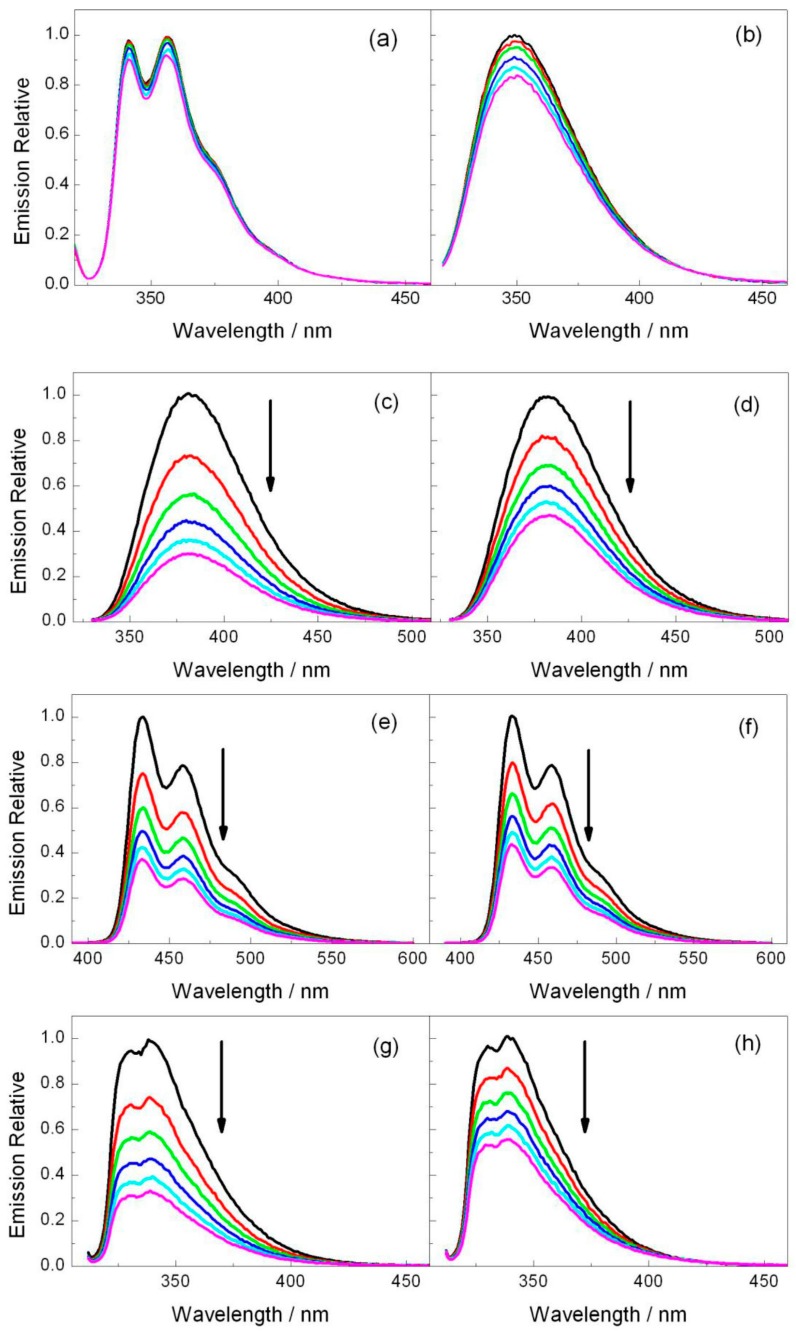
Fluorescence emission spectra for (**a**) CAR (λ_exc_ = 310 nm) and (**b**) DMA (λ_exc_ = 310 nm) in the presence of increasing amounts of **2b** (0–12.5 mM) as well as (**c**,**d**) DCN (λ_exc_ = 310 nm), (**e**,**f**) DCA (λ_exc_ = 375 nm), and (**g**,**h**) CNN (λ_exc_ = 310 nm) in the presence of increasing amounts of **2a** (left panel) or **2b** (right panel) (0–12.5 mM). The quencher concentration is 0 (black), 2.5 (red), 5 (green), 7.5 (blue), 10 (light blue) and 12.5 mM (pink).

**Figure 3:**
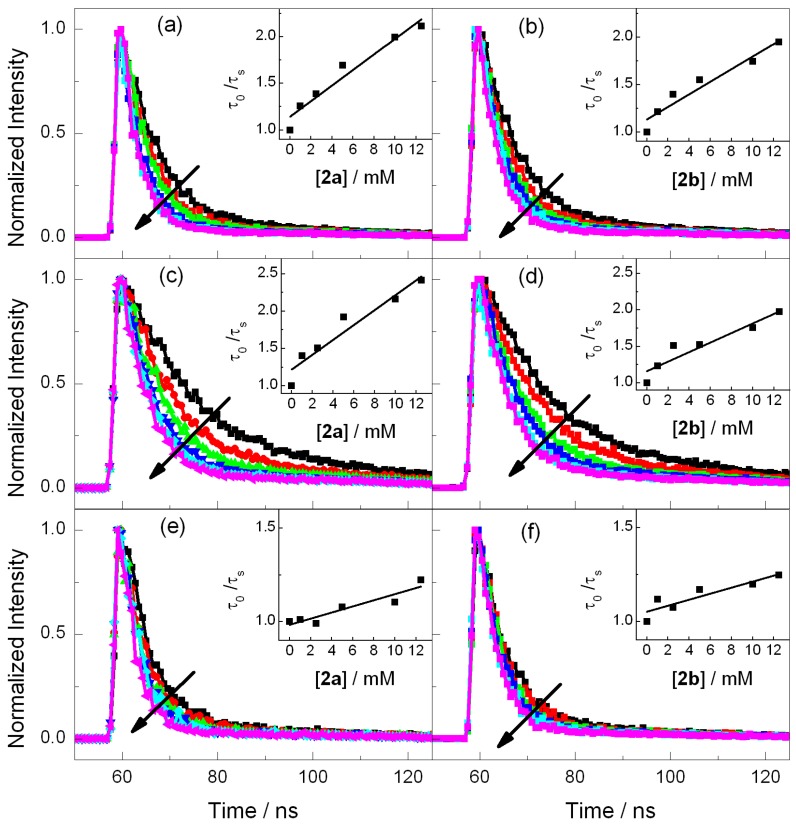
Fluorescence kinetic traces obtained for DCN (λ_exc_ = 310 nm) (**a**,**b**), DCA (λ_exc_ = 375 nm) (**c**,**d**) and CNN (λ_exc_ = 310 nm) (**e**,**f**) in the presence of increasing amounts of **2a** (left panel) or **2b** (right panel) from 0 to 12.5 mM. Inset: corresponding Stern Volmer plots. The quencher concentration is 0 (black), 2.5 (red), 5 (green), 7.5 (blue), 10 (light blue) and 12.5 mM (pink).

Steady-state fluorescence experiments were performed for the selected photosensitizers in order to evaluate the possibility of an electron transfer process with both azetidines. As stated above, electron donor and acceptor sensitizers were selected covering a large range of singlet excited state redox properties (see Table 1). They include DMA and CAR for the photoreductants and DCN, DCA and CNN for the photooxidants (Figure 1) [20,22]. Thus, acetonitrile solutions of the photosensitizer were prepared with an absorbance of ca. 0.15 at the excitation wavelength, which was fixed at λ_exc_ = 310 nm for DMA, CAR, DCN and CNN and λ_exc_ = 375 nm for DCA (see absorption spectra in Figure S6), and the fluorescence intensity was measured in the absence and in the presence of **2a** or **2b**. As observed in Figure 2, the emission spectra of CAR and DMA suffer only weak changes when azetidine is added, which signifies that electron injection is not an efficient process. By contrast, the photooxidant emissions underwent important quenching (Figure 2). In order to evaluate the efficiency of this oxidative electron transfer, time-resolved fluorescence was performed. Figure 3 shows the decays obtained for all the photosensitizers alone or in the presence of increasing amounts of compounds **2a** or **2b**. The singlet lifetime of all these photooxidants was shortened in the presence of azetidine derivatives. For example, the DCA lifetime reached values of 6.3 ns in the presence of 12.5 mM of **2a** (Figure 3c). Interestingly, this value was somewhat higher for **2b** (Figure 3d), being of ca. 7.2 ns. The bimolecular quenching rate constants (k_q_, Table 1) were determined through Stern-Volmer analysis (see Materials and Methods) by plotting the ratio τ_0_/τ, between the lifetime in the absence and in the presence of quencher, as a function of the quencher concentration [24]. The quenching process was more efficient as the reduction potential in the excited state (E*) increased, which supports an electron transfer mechanism from the azetidine to the excited PS. Indeed, energy transfer can be ruled out because the absorption bands of **2a** and **2b** are below 300 nm (see Figure S7). Thus, their singlet energies (E_S_) can be safely estimated to be >400 kJ·mol^−1^, that is more than 25 kJ·mol^−1^ higher than that of the highest E_S_ of the selected photosensitizer (Table 1).

Moreover, a significant difference of electron transfer efficiency was observed between **2a** and **2b** (see k_q_, Table 1), the *cis* isomer being a more efficient quencher than the *trans* **2b**. The two isomers have the same bonds and connectivity; however, they differ in the arrangement of the dihydroazauracil and cyclohexane rings. A similar stereodifferentiation has previously been reported for photoreduction of the dimethylthymine cyclobutane dimer *cis-syn* and *trans-syn* diastereoisomers [25]. The more difficult reduction of the *cis-syn* isomer was attributed to a stereoelectronic effect as the formed anion radical suffers an unfavorable charge-dipole interaction with the carbonyl in C_4_ of the other pyrimidine ring. Nevertheless, this explanation cannot be extended to **2a** and **2b** as the cyclohexane ring does not provide pronounced electronic effects, and the most likely explanation relies on different degrees of steric hindrance of the azetidine ring.

### 2.3. Electrochemistry

**Figure 4:**
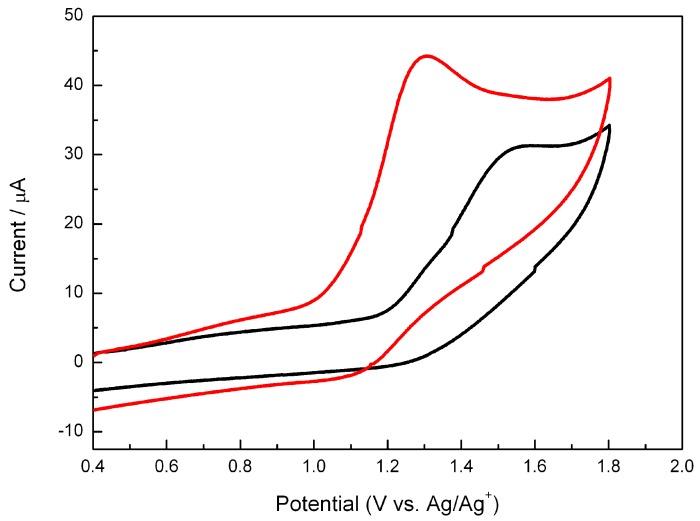
Cyclic voltammograms of **2a** (red line, 1 mM) and **2b** (black line, 1 mM) in acetonitrile using 0.1 M Bu_4_NClO_4_ as electrolyte. Scan rate: 0.5 V·s^−1^.

The different behavior observed between **2a** and **2b** must be connected with the redox properties of the two isomers. Thus, their electrochemical behavior was investigated by cyclic voltammetry using a three electrode standard configuration with two platinum wires as working and counter electrodes, and Ag/AgCl in saturated KCl as reference electrode. The cyclic voltammograms obtained at a scan rate of 0.5 V·s^−1^ are shown in Figure 4. A single wave was observed for both isomers, the oxidation being more difficult for **2b** than for **2a** as reflected by the more positive values of the peak potential (E_p_(**2a**) = 1.30 V and E_p_(**2b**) = 1.55 V). This behavior is similar to that described for oxetanes or thietanes, where removal of one electron leads to cleavage of the heterocyclic 4-membered ring [9,17]. Moreover, the easier oxidation of **2a** is in full agreement with the photochemical results described in the previous section that established a more efficient electron transfer for the *cis* isomer.

## 3. Materials and Methods

### 3.1. Chemicals

6-Azauracil, cyclohexene, CAR, DMA, DCN, DCA and CNN were purchased from Sigma-Aldrich (Madrid, Spain) and were used without further purification. Column chromatography was run with silica gel 60 A CC 35–70 μm as stationary phase, purchased from Merck (Madrid, Spain) and using analytical grade solvents obtained from Scharlau (Sentmenat, Spain). Acetonitrile HPLC grade (Scharlau) was used for fluorescence and electrochemical studies.

### 3.2. Instrumentation

NMR spectra were recorded on an AV 300 spectrometer (Bruker, Rivas-Vaciamadrid, Spain) at 296 K using CDCl_3_ as solvent. Chemical shifts are referenced to the solvent signal i.e., 7.26 ppm and 77.2 ppm for ^1^H-NMR and ^13^C-NMR, respectively.

HRMS were obtained by electrospray mass spectroscopy in the positive mode mode using a Xevo QTof MS apparatus (Waters Corp., Cerdanyola del Vallès, Spain). The ESI source was operated in positive ionization mode with the capillary voltage at 1.9 kV. The temperature of the source and desolvation was set at 80 °C and 400 °C, respectively. The cone and desolvation gas flows were 20 L·h^−1^ and 800 L·h^−1^, respectively. All data collected in Centroid mode were acquired using Masslynx™ software (V4.1 Waters Corp.). Leucine-enkephalin was used as the lock mass generating an [M + H]^+^ ion (*m*/*z* 556.2771) at a concentration of 250 pg/mL and flow rate of 50 μL/min to ensure accuracy during the MS analysis.

Steady-state fluorescence experiments were carried out on a PTI LPS-220B spectrofluorometer (Photon Technology International, Longjumeau, France). Time-resolved fluorescence measurements were performed with a EasyLife V spectrometer from OBB (Palaiseau, France), equipped with a pulsed LED (λ_exc_ 310 nm or 375 nm) as excitation source; residual excitation signal was filtered in emission by using a cut-off filter (50% transmission at 320 nm or 400 nm for the 310 or 375 nm excitation, respectively). The kinetic traces were fitted by one monoexponential decay function, using a deconvolution procedure to separate them from the lamp pulse profile. All experiments were performed in a quartz cuvette of 1 cm of optical path.

Cyclic voltammetry measurements were performed with a VersaSTAT 3 potentiostat (Princeton Applied Research, Algete-Madrid, Spain) and using a three electrode standard configuration with two platinum wires as working and counter electrodes, and Ag/AgCl in saturated KCl as reference electrode. Measurements were carried out on an acetonitrile solution of **2a** or **2b** (1 mM) with 0.1 M Bu_4_NClO_4_ as electrolyte at a scan rate of 0.5 V·s^−1^. All the solutions were previously purged with N_2_ for at least 15 min before the measurements. Ferrocene were taken as internal standard and measured potentials have been referenced to the E_1/2_ potential of the ferrocinium/ferrocene (Fc^+^/Fc) couple of 0.425 V in CH_3_CN [26].

### 3.3. Synthesis

*1,3-Dimethyl-6-azauracil* (**1**): 6-Azauracil (1.0 g, 8.84 mmol) was added to a suspension of NaH (0.5 g, 19.45 mmol) in anhydrous dimethylformamide (22 mL) at 0 °C. After stirring for 1 h at room temperature, the reaction mixture was treated with iodomethane (1.2 mL, 19.45 mmol) and stirred for 16 h. The solvent was removed, and the residue was purified by flash chromatography (silica gel, 100% dichloromethane) to give **1** (0.8 g, 70%) as a white solid powder. ^1^H-NMR (300 MHz, CDCl_3_) δ 7.36 (s, 1H), 3.63 (s, 3H), 3.33 ppm (s, 3H). ^13^C-NMR (75 MHz, CDCl_3_) δ 156.4 (CO), 149.1 (CO), 133.9 (CH), 39.8 (CH_3_), 27.0 ppm (CH_3_).

*1,3-Dimethyl-6-azauracilcyclohexene* (**2a**, **2b**). A solution of **1** (0.5 g, 3.54 mmol), acetone (10 mL) and cyclohexene (2.5 mL) in acetonitrile (30 mL) was placed in a Pyrex vessel (λ > 290 nm), purged with nitrogen and irradiated for 6 h using a medium pressure mercury lamp (750 W). The solvent was removed, and the residue was purified by flash chromatography (silica gel, 7% ethyl acetate/dichloromethane) to give two products as white solids.

**2a** (0.32 g, 41%): ^1^H-NMR (300 MHz, CDCl_3_) δ 4.33 (d, *J* = 9.0 Hz, 1H), 4.01 (apparent q, *J* = 8.5 Hz, 1H), 3.15 (s, 3H), 2.98 (s, 3H), 2.93 (m, 1H), 2.04–1.93 (m, 1H), 1.84–1.69 (m, 1H), 1.65–1.30 (m, 4H), 0.99–0.87 ppm (m, 2H). ^13^C-NMR (75 MHz, CDCl_3_) δ 170.0 (CO), 152.5 (CO), 66.0 (CH), 63.9 (CH), 36.2 (CH), 30.4 (CH_3_), 26.8 (CH_3_), 23.3 (CH_2_), 22.0 (CH_2_), 21.4 (CH_2_), 19.9 ppm (CH_2_). HRMS (ESI): *m*/*z* calcd for C_11_H_18_N_3_O_2_ [M + H]^+^ 224.1399, found 224.1407.

**2b** (0.28 g, 36%): ^1^H-NMR (300 MHz, CDCl_3_) δ 4.03–3.91 (m, 2H), 3.24 (s, 3H), 3.19 (s, 3H), 2.42 (q, *J* = 8.8 Hz, 1H), 2.20–1.58 (m, 5H), 1.49–1.31 (m, 1H), 1.16–0.96 ppm (m, 1H). ^13^C-NMR (75 MHz, CDCl_3_) δ 171.4 (CO), 151.9 (CO), 67.1 (CH), 66.9 (CH), 38.4 (CH), 33.0 (CH_3_), 28.1 (CH_2_), 27.4 (CH_2_), 27.1 (CH_3_), 22.2 (CH_2_), 20.2 (CH_2_). HRMS (ESI): *m*/*z* calcd for C_11_H_18_N_3_O_2_ [M + H]^+^ 224.1399, found 224.1392.

### 3.4. Fluorescence Quenching

The absorbance of the sensitizer for the fluorescence experiments was kept 0.15 at the excitation wavelength (λ_exc_ = 310 nm for CAR, DMA, DCN and CNN, or 375 nm for DCA). On the other hand, a stock solution of azetidine **2a** or **2b** (0.15 M) was prepared, so it was only necessary to add microliter volumes to the sample cell to obtain appropriate concentration of the quencher. The rate constants (k_q_) for the reaction were obtained from the Stern-Volmer plots [24] following Equation (1):

1/τ = 1/τ_0_ + k_q_ × [**2a**] or 1/τ = 1/τ_0_ + k_q_ × [**2b**] (1)

where τ_0_ is the lifetime of the photosensitizer in the absence of **2a** or **2b** and τ is the lifetime after addition of a quencher concentration [**2a**] or [**2b**].

## 4. Conclusions

The initial electron transfer step assumed to be involved in the repair of thymine-cytosine (6-4) photoproducts by photolyases has been investigated here by using azetidines **2a** or **2b** and photosensitizers DMA, CAR, DCN, DCA and CNN as model compounds. The fluorescence of DMA and CAR is hardly quenched by **2a** or **2b**, indicating that the photoreductive pathway is not efficient in the corresponding donor-acceptor pairs. Conversely, an efficient fluorescence quenching is observed for the cyanoaromatics DCN, DCA and CNN, both in steady-state and time-resolved experiments. The dynamic quenching supports an efficient oxidative electron transfer, which is faster for the *cis* isomer **2a**. Electrochemical studies are in agreement with these results, showing that oxidation of **2a** is easier due to its more favorable redox potential.

## Figures and Tables

**Table 1:** Photophysical properties of the selected photosensitizers and their quenching rate constants by **2a** and **2b**.

PS	/ns	E*/V	E_S_ (kJ·mol^−1^)	k_q_ (2a)/10^9^ M^−1^·s^−1^	k_q_ (2b)/10^9^ M^−1^·s^−1^
DMA	1.5	−3.0 (PS**^+^**^●^/PS*) ^1^	363	N.D. ^3^	N.D. ^3^
CAR	7.4	−2.5 (PS**^+^**^●^/PS*) ^1^	338	N.D. ^3^	N.D. ^3^
DCN	7.5	2.4 (PS*/PS^●**−**^) ^2^	362	10	7.7
DCA	12.5	1.8 (PS*/PS^●**−**^) ^2^	276	6.4	4.6
CNN	5.5	1.4 (PS*/PS^●**−**^) ^2^	374	3.2	2.6

^1^ From reference [20], vs. Ag/AgCl (saturated KCl). ^2^ From reference [22], vs. Ag/AgNO_3_. ^3^ N.D.: Not determined.

## Supplementary Materials

Supplementary materials can be accessed at: http://www.mdpi.com/1420-3049/21/12/1683/s1.
