# Supplementary material for: Stereoselective Fluorescence Quenching in the Electron Transfer Photooxidation of Nucleobase-Related Azetidines by Cyanoaromatics
Source: Molecules. 2016 Dec 7;21(12):1683. doi: 10.3390/molecules21121683 (PMC6273614; doi:10.3390/molecules21121683)
Supplement: Supplementary file 1 [file molecules-21-01683-s001.pdf]

# Supplementary Materials: Stereoselective Fluorescence Quenching in the Electron Transfer Photooxidation of Nucleobase-Related Azetidines by Cyanoaromatics

**Ana B. Fraga-Timiraos, Gemma M. Rodríguez-Muñiz, Vicente Peiro-Penalba, Miguel A. Miranda and Virginie Lhiaubet-Vallet**

**Table of Contents**

Figures S1–S3: $^{1}$H, DEPT and $^{13}$C-NMR spectra of **1**, **2a** and **2b** Pages S2–S3

Figure S4: NOESY spectra of **2a** and **2b** Page S4

Figure S5: HQMC spectra of **2a** and **2b** Page S5

Figure S6: UV absorption spectra of the selected photosensitizers Page S6

Figure S7: UV absorption spectra of **2a** and **2b** Page S7

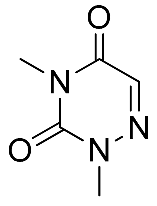


**1**

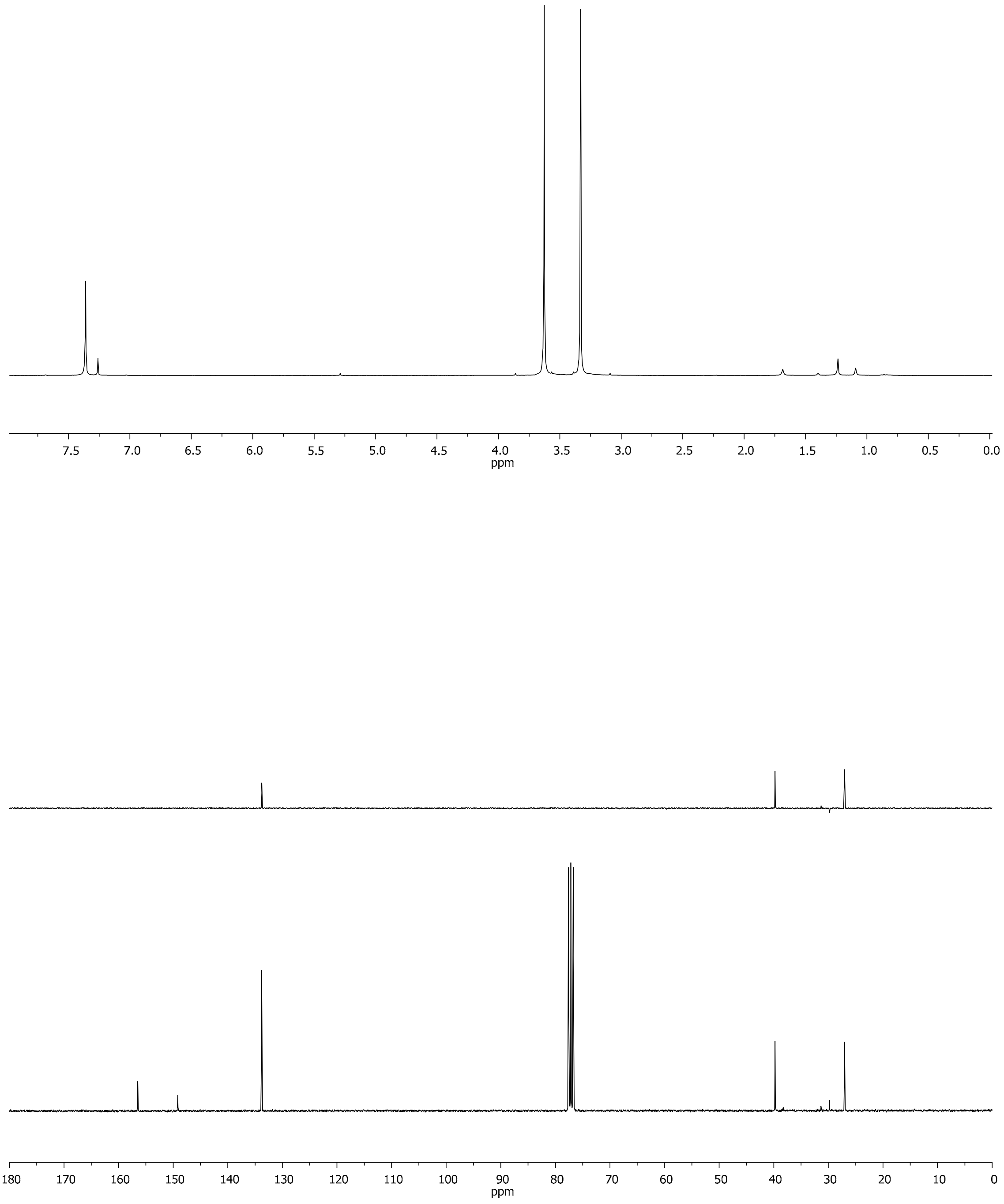


**Figure S1.** $^{1}H$, DEPT and $^{13}C$ NMR spectra of compound **1**.

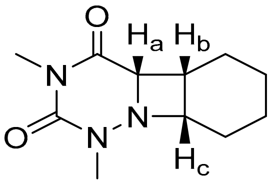


**2a**

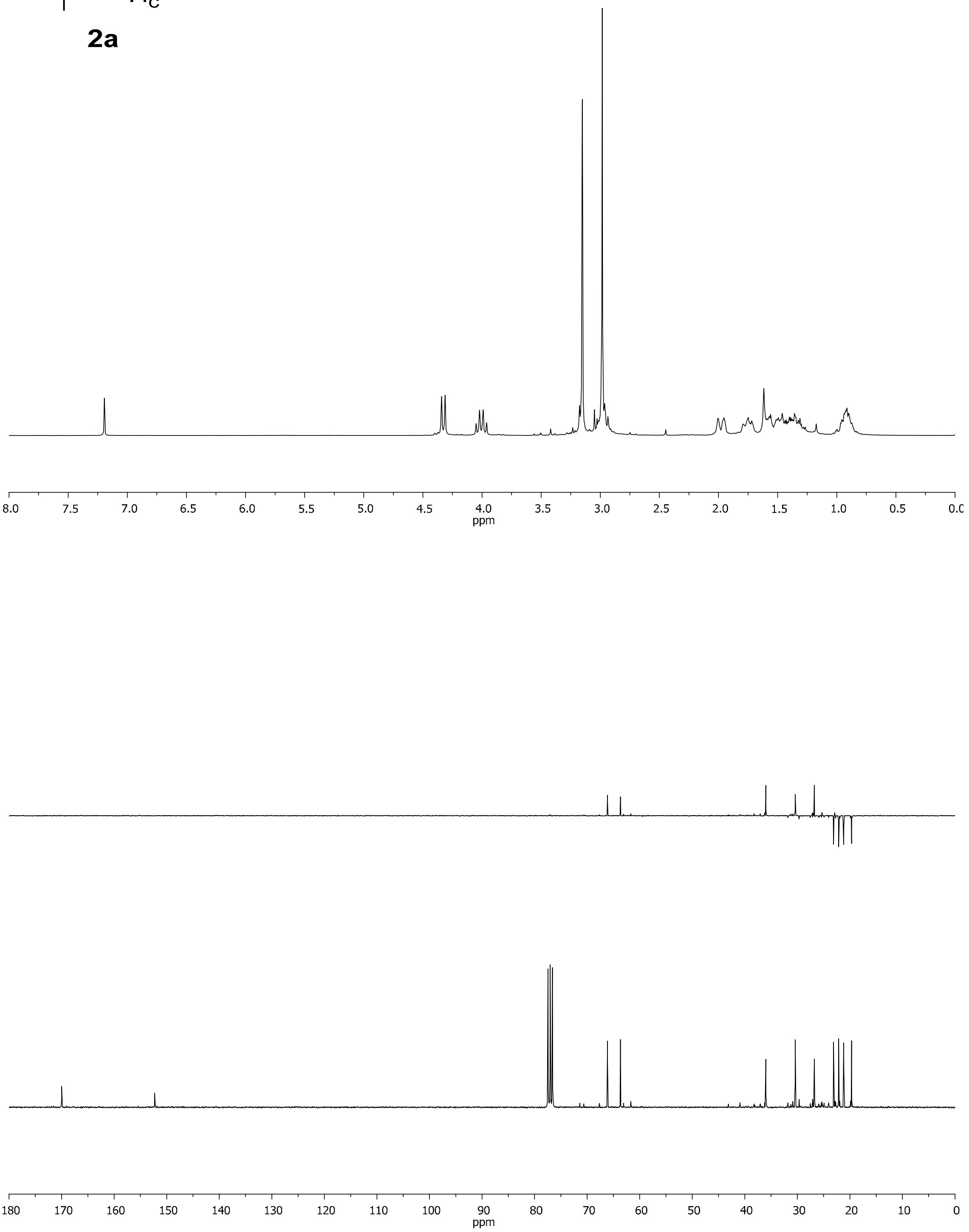


**Figure S2.** $^{1}H$, DEPT and $^{13}C$ NMR spectra of compound **2a**.

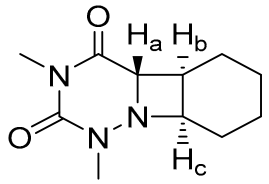


**2b**

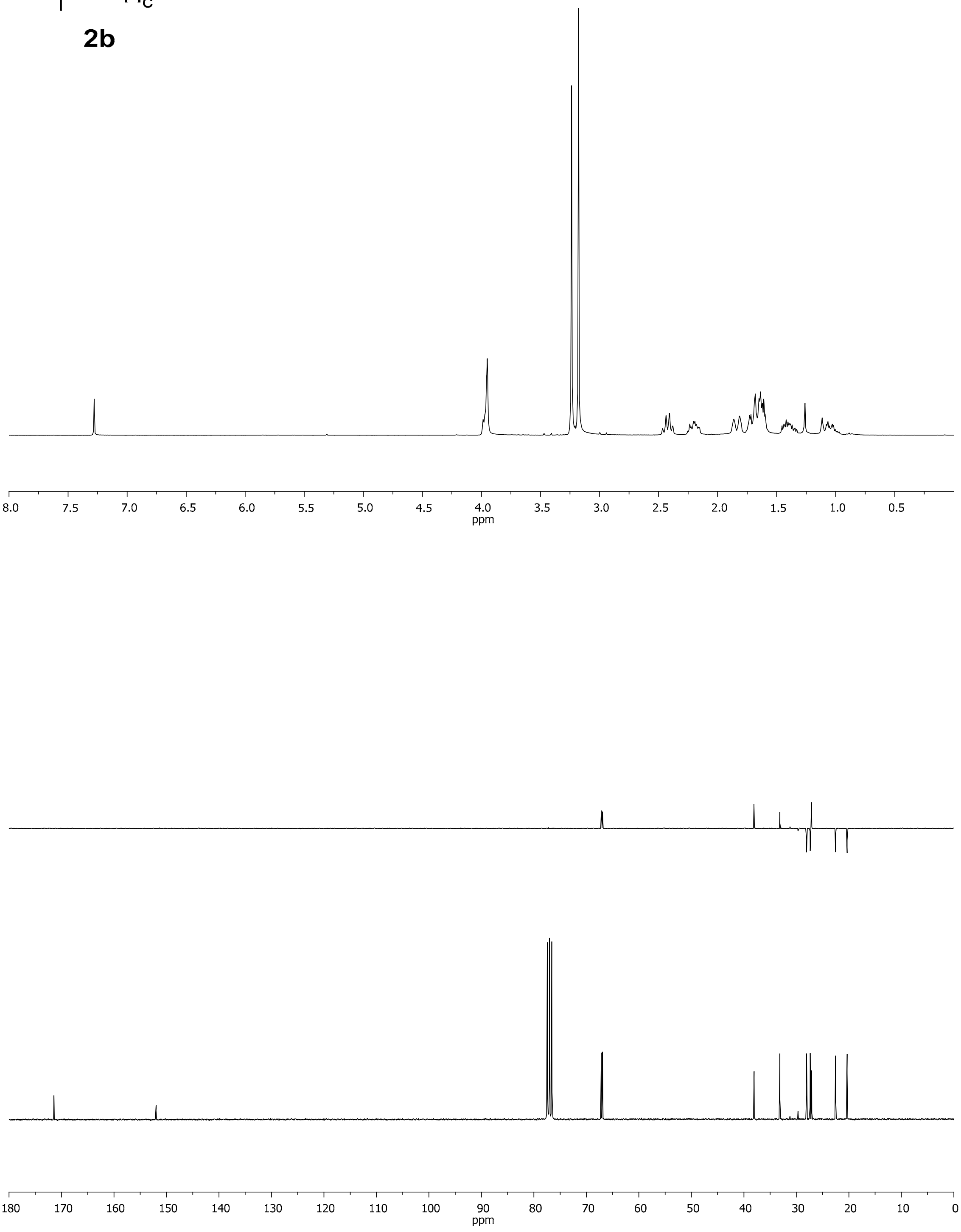


**Figure S3.** $^{1}H$, DEPT and $^{13}C$ NMR spectra of compound **2b**.

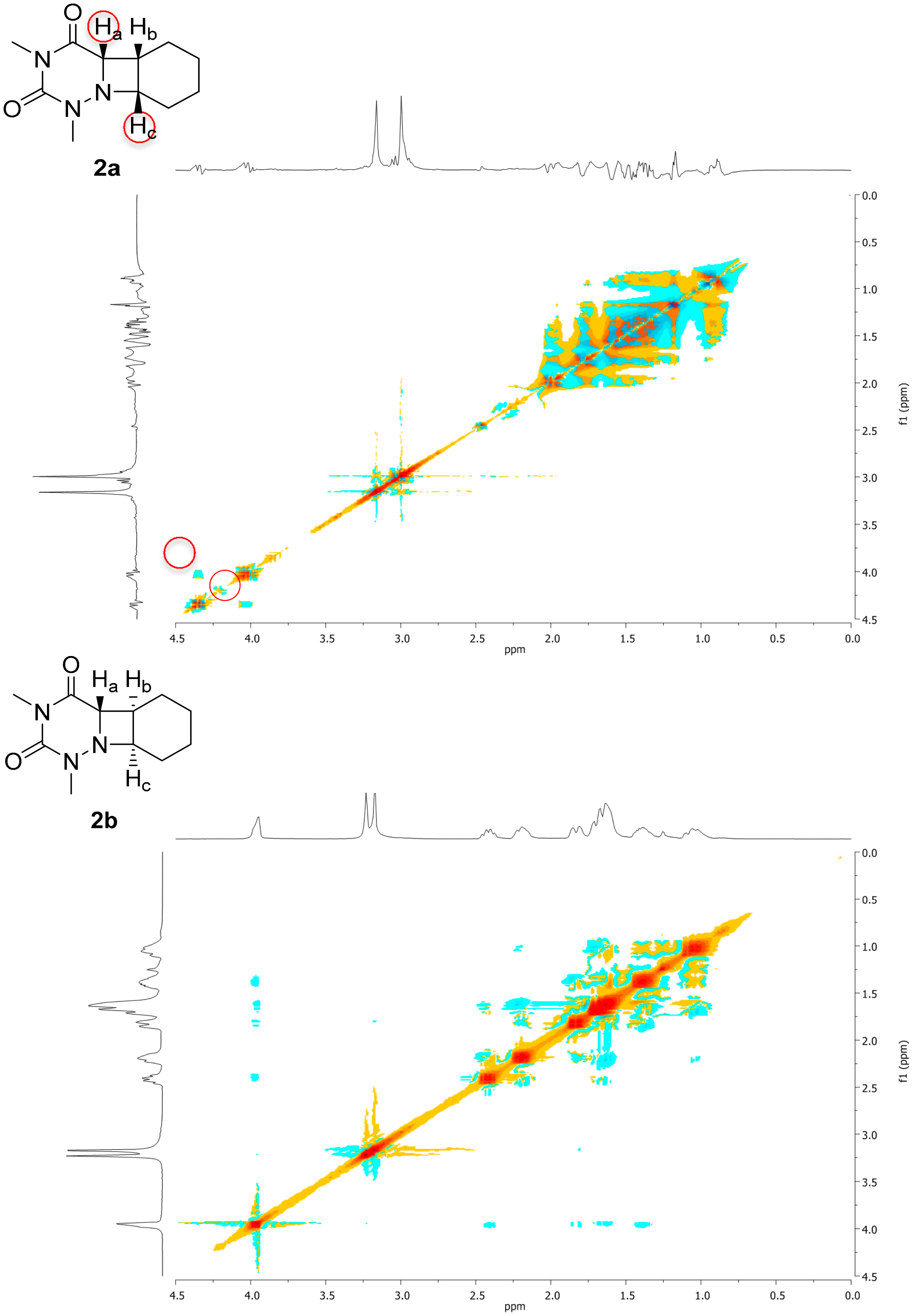


**Figure S4.** NOESY spectra of compounds **2a** and **2b**.

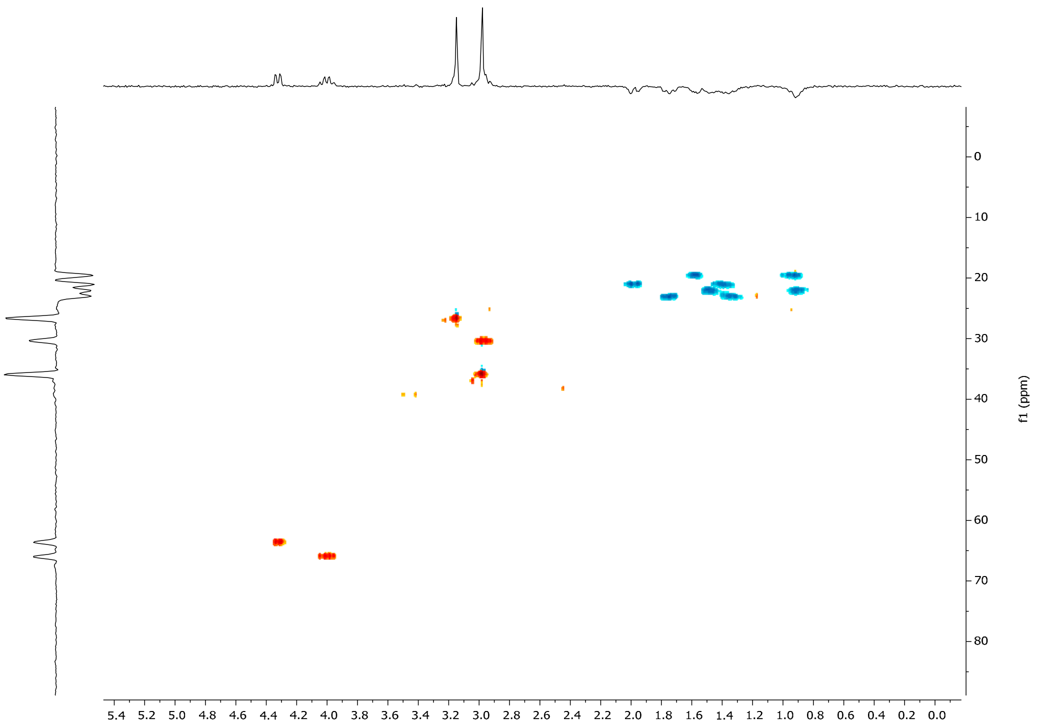


**Figure S5.** HMQC spectra of compounds **2a** (top) and **2b** (bottom).

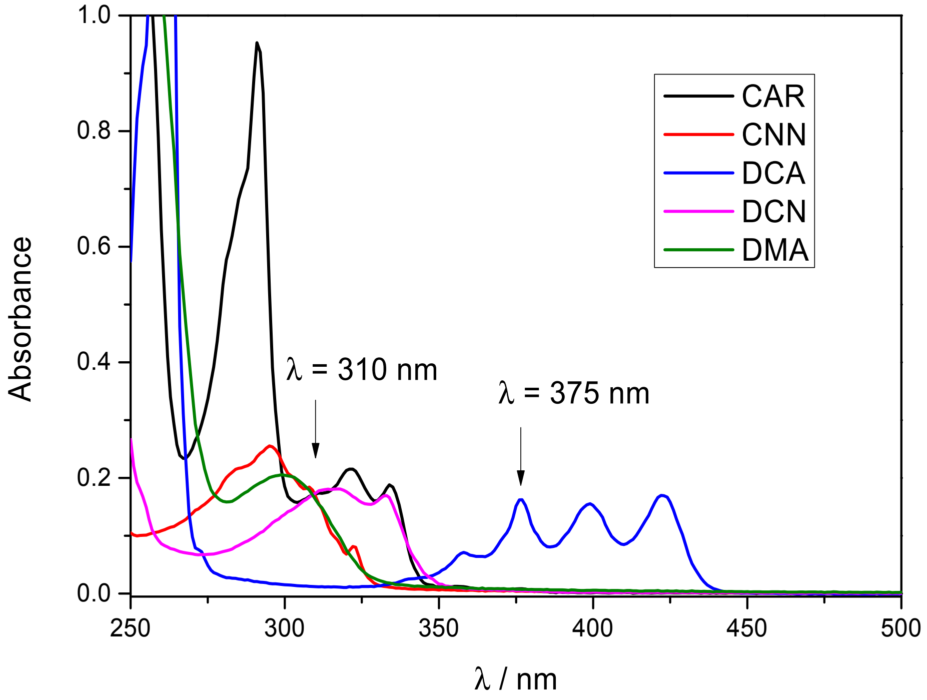


**Figure S6.** UV Absorption spectra of the selected photosensitizers in acetonitrile.

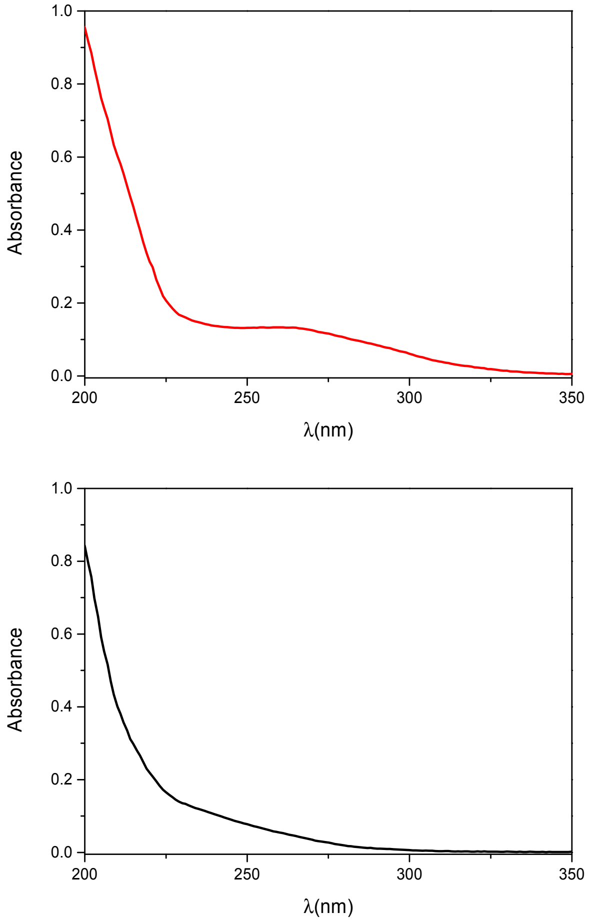


**Figure S7.** UV absorption spectra of compounds **2a** (top) and **2b** (bottom).
